# Inhibition of GSK-3β Rescues the Impairments in Bone Formation and Mechanical Properties Associated with Fracture Healing in Osteoblast Selective Connexin 43 Deficient Mice

**DOI:** 10.1371/journal.pone.0081399

**Published:** 2013-11-08

**Authors:** Alayna E. Loiselle, Shane A. J. Lloyd, Emmanuel M. Paul, Gregory S. Lewis, Henry J. Donahue

**Affiliations:** Division of Musculoskeletal Sciences, Department of Orthopaedics and Rehabilitation, Penn State College of Medicine, Hershey, Pennsylvania, United States of America; Georgia Regents University, United States of America

## Abstract

Connexin 43 (Cx43) is the most abundant gap junction protein in bone and is required for osteoblastic differentiation and bone homeostasis. During fracture healing, Cx43 is abundantly expressed in osteoblasts and osteocytes, while Cx43 deficiency impairs bone formation and healing. In the present study we selectively deleted Cx43 in the osteoblastic lineage from immature osteoblasts through osteocytes and tested the hypothesis that Cx43 deficiency results in delayed osteoblastic differentiation and impaired restoration of biomechanical properties due to attenuated β-catenin expression relative to wild type littermates. Here we show that Cx43 deficiency results in alterations in the mineralization and remodeling phases of healing. In Cx43 deficient fractures the mineralization phase is marked by delayed expression of osteogenic genes. Additionally, the decrease in the RankL/ Opg ratio, osteoclast number and osteoclast size suggest decreased osteoclast bone resorption and remodeling. These changes in healing result in functional deficits as shown by a decrease in ultimate torque at failure. Consistent with these impairments in healing, β-catenin expression is attenuated in Cx43 deficient fractures at 14 and 21 days, while Sclerostin (*Sost*) expression, a negative regulator of bone formation is increased in Cx43cKO fractures at 21 days, as is GSK-3β, a key component of the β-catenin proteasomal degradation complex. Furthermore, we show that alterations in healing in Cx43 deficient fractures can be rescued by inhibiting GSK-3β activity using Lithium Chloride (LiCl). Treatment of Cx43 deficient mice with LiCl restores both normal bone formation and mechanical properties relative to LiCl treated WT fractures. This study suggests that Cx43 is a potential therapeutic target to enhance fracture healing and identifies a previously unknown role for Cx43 in regulating β-catenin expression and thus bone formation during fracture repair.

## Introduction

Connexin 43 (Cx43) is encoded by the *Gja1* gene and is the most highly expressed gap junction protein in bone [1]. Gap junctions are formed by the docking of Connexons, or hemichannels, on the surface of adjacent cells and each Connexon is composed of six connexin subunits [2]. Functional gap junctions permit the passage of small molecules (less than 1Kd) between cells. In bone, the transmission of signals, including mechanical signals between the bone cell network [3-5] are important in the bone response to loading and unloading [6-11]. Gap junctions composed of Cx43 facilitate communication between osteoblasts and osteocytes as well as between the osteocytic network [12]. Cx43 is required for osteoblastic proliferation [13], survival [14] and differentiation [15-17]. In addition to regulating osteoblast maturation *in vitro*, Cx43 plays an important role in bone homeostasis *in vivo* as demonstrated by the osteopenic phenotype that is associated with several models of Cx43 deficiency in bone [10,18,19]. Global alterations in Cx43 expression result in perinatal lethality due to neural tube defects and patent ductus arteriosus upon complete loss of Cx43 or systemic overexpression of Cx43 [20,21]. The development of phenotypes associated with systemic changes in Cx43 expression necessitates the use of conditional deletion constructs to study the functions of Cx43 in bone *in vivo* post-natally. In addition, loss of Cx43 in mature osteoblasts and osteocytes, by means of the human Osteocalcin promoter driven Cre, results in impaired fracture healing due to defects in bone formation and remodeling [22]. In the current study we have generated mice with Cx43 deficiency in the osteoblastic lineage using Col1-Cre to delete Cx43 in the osteoblastic lineage from immature osteoblasts through osteocytes. We tested the hypothesis that Cx43 deficiency results in delayed osteoblastic differentiation and impaired restoration of biomechanical properties due to attenuated β-catenin expression relative to wild type littermates. We further propose that β-catenin expression is reduced as a result of antagonism by Sclerostin and/ or increased GSK-3β activity, and that the fracture healing phenotype in Cx43 deficient mice could be rescued by restoring β-catenin expression through inhibition of GSK-3-β activity with Lithium Chloride (LiCl) treatment.

 β-catenin signaling is activated in the presence of Wnt ligands, and nuclear translocation of β-catenin results in transcription of osteogenic genes in cooperation with TCF/Lef1. In the absence of Wnt ligands, or through inhibition of Wnt signaling, including signaling antagonists such as Sclerostin, β-catenin undergoes proteasomal degradation through a signaling complex composed of GSK-3β, adenomatous polyposis coli (APC) and Axin [23-25]. Stabilization of β-catenin differentially affects fracture healing depending on the specific stage of healing in which activation occurs. Stabilization of β-catenin prior to fracture and during the initial inflammatory stage inhibits the differentiation of mesenchymal stem cells (MSCs) to chondrocytes and osteoblasts and leads to a persistence of undifferentiated mesenchymal tissue. In contrast, activation of β-catenin after MSCs have committed to the chondrogenic or osteogenic lineage improves healing and results in an increase in bone formation [26]. Here we show that deletion of Cx43 in osteoblasts and osteocytes results in decreased β-catenin expression, which coincides with increased Sclerostin expression and GSK-3β activity. This study suggests a previously unknown role for Cx43 in regulating β-catenin expression during fracture repair by modulating GSK-3β activity; a finding that is particularly important given the distinct effects β-catenin activation can have during different phases of healing. 

These data suggest that temporally and spatially targeted overexpression of Cx43 could enhance fracture healing by promoting β-catenin expression. 

## Methods

### Animal Model

This study was carried out in strict accordance with the recommendations in the Guide for the Care and Use of Laboratory Animals of the National Institutes of Health. The protocol was approved by the Institutional Animal Care and Use Committee (IACUC) at Penn State College of Medicine (Protocol Number: 94-120). All surgery was performed under Ketamine/ Xylazine anesthesia, and all efforts were made to minimize suffering. 

A closed femur fracture was produced by three-point bending [27] in 10-12 week old female wild type (WT; Col1-Cre^-^ Cx43^flox/flox^) and Cx43 conditional knockout mice (Cx43cKO; Col1-Cre^+^ Cx43^flox/flox^). Fractures were stabilized with a 25g intramedullary pin. Cx43cKO mice were generated by crossing Col1-Cre (2.3Kb) mice [28] (obtained from Mutant Mouse Regional Resource Center (MMRRC) at UC Davis) to Cx43 flox/flox mice (from Dr. Roberto Civitelli) [29]. This cross resulted in selective deletion of Cx43 in the osteoblastic lineage beginning in immature osteoblasts through osteocytes. Healing was assessed radiographically each week and femurs were harvested between three and 35 days post-fracture. For LiCl treatment, 0.6M LiCl was diluted in tap water to 0.02M, which resulted in an approximate daily dose of 200mg/kg/day as previously described [26]. LiCl treatment was initiated at day 4 post-fracture, a time-point that has been reported to result in bone formation rather than a preponderance of undifferentiated mesenchyme in the fracture callus [26].

### RNA Extraction and qPCR

The fracture callus and approximately 1mm of cortical bone on each side of the callus was harvested and RNA extracted using TRIzol reagent (Invitrogen, Grand Island, NY). RNA from 3-specimens/ genotype/ time-point was pooled. cDNA was reverse transcribed using iScript (Bio-Rad, Hercules, CA) and relative expression of specific genes was analyzed by Real-Time RT-PCR using SYBR Green (Qiagen, Germantown, MD) and specific primers for mouse *Gja1*, *Col1a1*, Osteocalcin (*Bglap*)*, Bmp2*, RankL (*Tnsfs11*), Opg (*Tnsfs11b*) , *β-catenin, Sost* and *GSK-3β*. Gene expression was normalized to β-actin (*Actb*) and expression in WT day 3 fractures. 

### Western Blotting

Following sacrifice, the fracture callus (flushed of marrow) was isolated from WT and Cx43cKO mice at 21 days post-fracture. Protein was lysed using NP-40 lysis buffer and lysates were run on 4-20% Tris-Glycine gels. Blots were probed with the following antibodies: GSK-3β (Cell signaling #9315; 1:1000), Phospho-GSK-3β (Ser9)(Cell Signaling #9336; 1:500), Phospho-GSK-3β (Tyr 216) (Santa Cruz #sc-135653; 1:500) and GAPDH (Sigma # G8795; 1:10000). Densitometry analysis was conducted using NIH Image J software (rsbweb.nih.gov/ij/). Total GSK-3β and Phospho-GSK-3β were normalized to GAPDH and data are presented as normalized phospho-GSK-3β/ normalized GSK-3β.

### Histology

Femurs were harvested and fixed in 10% neutral buffered formalin for three days. Following de-calcification, tissues were processed and embedded in paraffin. 5-micron sections were used for TRAP staining of osteoclasts or immunohistochemistry. 

### TRAP Staining and Osteoclast Quantification

Tissue sections were stained with TRAP (tartrate resistant acid phosphatase) as previously described [30]. Following staining, the number of multinucleated (≥ 3 nuclei) TRAP^+^ osteoclasts were analyzed in a 1600 (height) x 2800 (width) micron area centered in the middle of the fracture callus. The number and size of TRAP^+^ osteoclasts were measured using BioQuant Osteo software (v12.5.60, BIOQUANT Image Analysis Corporation; Nashville, TN, USA) from 2 sections per specimen, 4-specimens/ genotype/ time-point.

### Immunohistochemistry

Paraffin sections were de-waxed, dehydrated, and underwent antigen retrieval using sodium citrate buffer (pH 6.0). Sections were probed with anti-β-Catenin antibody (#9562, Cell Signal, Danvers, MA), or anti-Sclerostin antibody (#AF1589, R&D Systems, Minneapolis, MN), followed by goat anti-rabbit (β-catenin), or rabbit anti-goat (Sclerostin) secondary antibody (Vector Labs, Burlingame, CA). Staining was visualized with DAB chromogen (Invitrogen) and sections were counterstained with Hematoxylin. Negative control sections underwent the same procedure but were probed with anti-rabbit IgG (# 3900S, Cell Signaling) or anti-goat IgG (#I-5000, Vector Labs). To count the number of Sclerostin^+^ osteocytes in different areas of bone, the cortical bone was segmented into three 1500 micron length sections and the number of Sclerostin^+^, viable (Sclerostin-), and empty lacunae were counted in these segments (proximal/distal cortical bone, the cortical bone adjacent to the fracture callus) (Figure S2A) and in a 2100 x 900 micron area of the fracture callus using BioQuant Osteo software. The percentage of Sclerostin^+^ osteocytes was calculated as the number of Sclerostin^+^ osteocytes/ total osteocytes (Sclerostin^+^, Sclerostin^-^, empty lacunae), while % empty lacunae was calculated as empty lacunae/ total lacunae. Osteocytes were counted in 3-specimens/ genotype/ time-point.

### Dynamic Histomorphometry

To determine changes in the rate of bone formation, mice were injected with 10mg/kg Calcein (in 2% sodium bicarbonate) 11 days prior to sacrifice and 30mg/kg Alizarin Red (in phosphate buffered saline; PBS) 4 days prior to sacrifice. Following harvest and removal of the intramedullary pin, specimens were dehydrated in sequential ethanols from 70-100%, and embedded in Osteo-Bed Bone Resin (Poly Sciences, Warrington, PA). Specimens were then cut with a low-speed diamond saw to a thickness of 100-microns, ground and polished using lapping films (3M, St. Paul, MN) to a thickness of 50-microns as previously described [10]. Axial sections were cut at the mid-point of the callus, and 2 sections/specimen were obtained from 4-specimens/ genotype/ time-point. Images were obtained at 4x magnification using a Leica microscope with a UV light source with red and green filters (Leica, Bannockburn, IL, USA). BioQuant Osteo software was used to measure the following raw data parameters from areas of new bone formation: single label surface perimeter (sL.Pm), double label surface perimeter (dL.Pm), double label area (dL.Ar) and Bone Perimeter (B.Pm). These parameters were used to calculate Mineral Apposition Rate (MAR= dL.Ar/dL.Pm/7 days; μm/day); Mineralizing surface/ bone surface (MS/BS= [1/2sL.Pm + dL.Pm]/B.Pm x 100; %) and Bone formation rate (BRF/BS= MAR x MS/BS x 3.65; μm^3^/μm^2^/year)[31].

### Micro CT

Following harvest, specimens were scanned with a VivaCT 40 Scanner (Scanco Medical AG, Brüttisellen, Switzerland) at high resolution with a voxel size of 10.5 μM. The reconstructed images were segmented and analyzed using the Image Processing Library supplied by the manufacturer. Micro CT analysis was completed between the first and last slice that had callus formation, resulting in an ~800-slice segment of bone for analysis. Bone volume (BV), total volume (TV) and BV/TV calculations included only callus tissue as images were segmented to exclude the cortical bone and marrow cavity. Specimens were analyzed at a threshold of 250, which includes moderate and high-density mineralized tissue, but excludes soft tissue and non-calcified cartilage. 

### Biomechanical Torsion Testing

After sacrifice, intramedullary pins were removed and femurs and were potted in Dynalite automotive body filler (3M, St. Paul, MN). Specimens were rehydrated in PBS and tested in torsion at a rate of 1°/sec until failure using a 177N-mm load cell. Torsional rigidity was calculated as the slope of the linear region of the curve of torque (N*mm) versus rotational deformation (Radians). Rotational deformation was normalized to gage length (mm). 

### Statistical Analysis

All data are presented ± standard error of the mean. Statistical evaluation was performed using a two-way Analysis of Variance (ANOVA) with post-hoc Bonferroni’s test to identify significant differences with respect to both genotype and time post-fracture.

## Results

### Cx43 is expressed in osteoblasts and osteocytes during fracture healing

**Figure 1:**
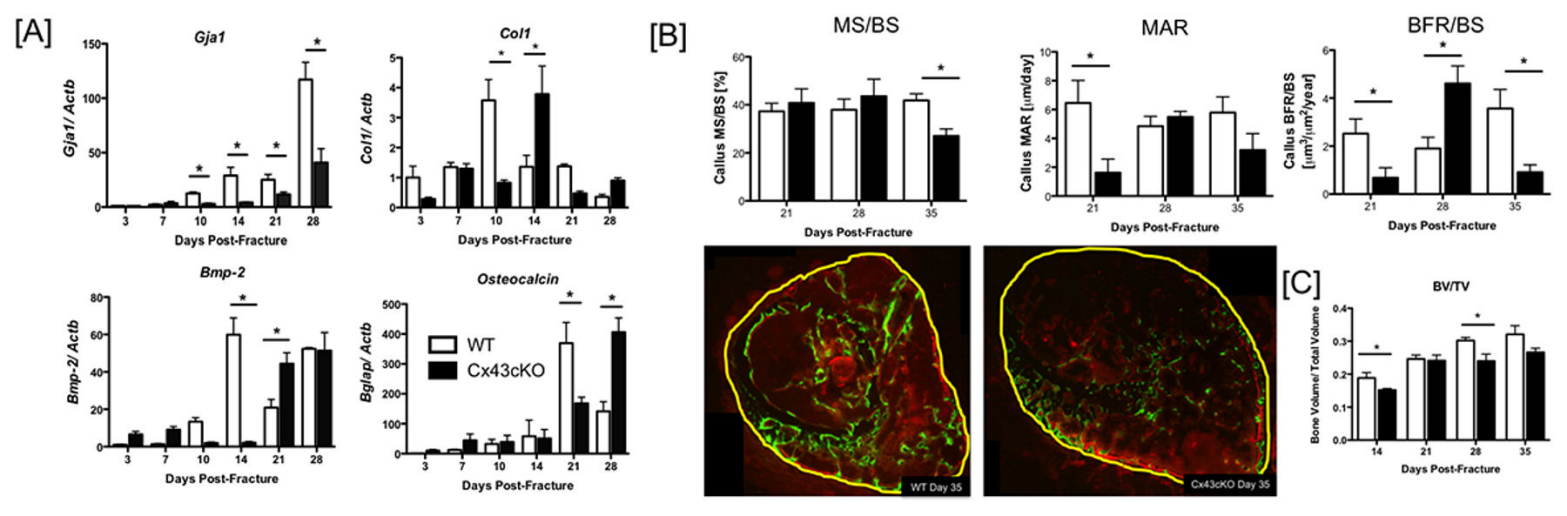
Delayed osteoblast maturation and bone formation in Cx43cKO fractures. [A] Expression of *Gja1*, *Col1a1*, osteocalcin and *Bmp2* in the fracture callus of WT and Cx43cKO mice between 3 and 28 days post-fracture. RNA was extracted from the fracture callus and pooled from 3 specimens/ genotype/ time-point. (*) indicates p<0.05 between genotypes at a given time-point. [B] Dynamic histomorphometry analysis of WT and Cx43cKO fractures between 14-35 days. Specimens were labeled with Calcein and Alizarin Red, with a 7-day labeling period and Mineral Apposition Rate (MAR), Bone Formation Rate (BFR/BS) and Mineralizing surface/ bone surface (MS/BS) were calculated. (*) indicates p<0.05, N=3-5 specimens/ genotype/ time-point. Representative images of WT and Cx43cKO fractures labeled with Calcein (green) and Alizarin Red (red) and harvested at day 35 post-fracture. Yellow line corresponds to the perimeter of the callus. [C] Bone volume/ total volume (BV/TV) of WT and Cx43cKO fractures between 14-35 days. (*) indicates p<0.05 between WT and Cx43cKO fractures at a given time-point.

*Gja1* expression was significantly elevated over WT day 3 expression levels beginning at 10 days post-fracture in WT mice (12.5-fold, p<0.001). During the time-course examined, peak expression of *Gja1* occurred at 28 days in WT fractures (117-fold). *Gja1* expression was significantly decreased in Cx43cKO fractures relative to WT between 10 and 28 days (Cx43cKO Day 28: 40.8-fold, p<0.0001) (Figure 1A). 

### Delayed Osteoblast Maturation in Cx43cKO fractures

Peak expression of Collagen I (*Col1a1*), a marker of immature osteoblasts, occurred at 10 days post-fracture in WT mice with a 3.57-fold increase over WT day 3 expression, and a significant increase relative to Cx43cKO fractures (0.82-fold, p<0.001). Peak *Col1a1* expression was delayed until 14 days in Cx43cKO fractures (3.78-fold vs. WT day 3 expression), but was significantly increased relative to WT (1.36-fold; p<0.001) at this time. *Bmp2*, a key regulator of osteoblast differentiation, was significantly increased in WT fractures (59.9-fold) relative to Cx43cKO (2.12-fold, p<0.001) at 14 days. This represented the time of maximal expression of *Bmp2* in WT. In contrast, peak expression of *Bmp2* was delayed until 21 days in Cx43cKO fractures (44.3-fold), with a significant increase relative to WT (20.8-fold, p<0.01). No difference in *Bmp2* expression was observed at 28 days post-fracture. Expression of osteocalcin (*Bglap*), a marker of mature osteoblasts, was also delayed in Cx43cKO fractures relative to WT. Peak osteocalcin expression occurred at 21 days in WT fractures (369-fold) and was significantly increased relative to Cx43cKO fracture (167-fold, p<0.01) at this time. Peak osteocalcin expression occurred at 28 days in Cx43cKO fractures (405-fold), which was significantly increased relative to WT (141-fold, p<0.0001) at this time (Figure 1A).

### Cx43 deficiency delays bone formation

To determine if changes in bone formation were the result of changes in the rate of bone formation, Calcein/ Alizarin Red labeled sections were analyzed. At 21 days, the mineral apposition rate (MAR) was significantly decreased in Cx43cKO fractures (1.62 ± 0.93 μm/day) relative to WT (6.45 ± 1.57 μm/day, p=0.038), however at 28 and 35 days, MAR was not significantly different between groups (p>0.05). Mineralizing surface/ bone surface (MS/BS) was not significantly different between WT and Cx43cKO fractures at 21 (WT: 37.30 ± 3.3%, Cx43cKO: 40.77 ± 5.8%, p=0.6) or 28 days (WT: 37.95 ± 4.4%, Cx43cKO: 43.57 ± 7.1; p=0.34). At 35 days MS/BS was significantly decreased in Cx43cKO fractures (27.0 ± 2.9%) relative to WT (41.8 ± 2.7 %, p=0.01). Bone Formation Rate (BFR/BS) was significantly increased in WT fractures (Day 21: 2.52 ± 0.61 μm^3^/μm^2^/ year) relative to Cx43cKO at 21 (0.68 ± 0.41 μm^3^/μm^2^/ year; p=0.047) and 35 days (Day 35-WT: 3.56 ± 0.79; Cx43cKO: 0.91 ± 0.31; p= 0.01). At 28 days BFR/BS was significantly increased in Cx43cKO fractures (4.61 ± 0.72 μm^3^/μm^2^/ year) relative to WT (1.90 ± 0.46 μm^3^/μm^2^/ year, p=0.013) (Figure 1B). 

### Decreased Bone Volume fraction in Cx43cKO fractures

To quantify changes in bone formation in the callus, callus bone volume (BV) and total callus volume (TV) were calculated. BV was not significantly different between WT and Cx43cKO fractures between 14 and 28 days post-fracture. Peak BV occurred at 14 days in WT fractures (WT: 6.47 ± 0.51, Cx43cKO: 5.64 ± 0.28, p=0.15) and progressively decreased over time (Figure S1A). In Cx43cKO fractures, peak BV occurred at 35 days with a significant increase relative to WT (WT: 3.15 ± 0.9, Cx43cKO: 6.53 ± 2.03, p=0.006). TV, a measure of mineralized and un-mineralized tissue in the callus was increased in Cx43cKO fractures at 14 and 35 days relative to WT fractures (Day 14-WT: 30.81 ± 2.33, Cx43cKO: 39.95 ± 2.52, p=0.016) (Figure S1B). TV was significantly decreased at 21 days relative to day 14 in WT and Cx43cKO fractures, although WT and Cx43cKO fractures were not significantly different than each other at this time (WT: 15.21 ± 2.44, Cx43cKO: 18.29 ± 2.96, p= 0.44). By 28 days, there was no difference in TV between WT (17.43 ± 1.18) and Cx43cKO fractures (21.16 ± 2.98, p=0.3). At day 35, TV was further decreased in WT and was significantly decreased compared to Cx43cKO (WT: 10.67 ± 1.5, Cx43KO: 25.11 ± 9.01, p=0.012). BV/TV (i.e. the proportion of the callus that is mineralized) was significantly decreased in Cx43cKO fractures at 14 days relative to WT (WT:0.188 ± 0.01, Cx43cKO: 0.151 ± 0.005, p=0.005), but was not different between WT and Cx43cKO at 21 days (p=0.82). BV/TV continued to increase at 28 days, and there was a significant increase in WT (0.30 ± 0.01) relative to Cx43cKO (0.24 ± 0.015, p=0.007). Peak BV/TV occurred at 35 days in both WT and Cx43cKO with no significant difference between groups (WT: 0.32 ± 0.05, Cx43cKO: 0.266 ± 0.03, p=0.064) (Figure 1C). 

### Impaired Osteoclastogenesis in Cx43cKO fractures

**Figure 2:**
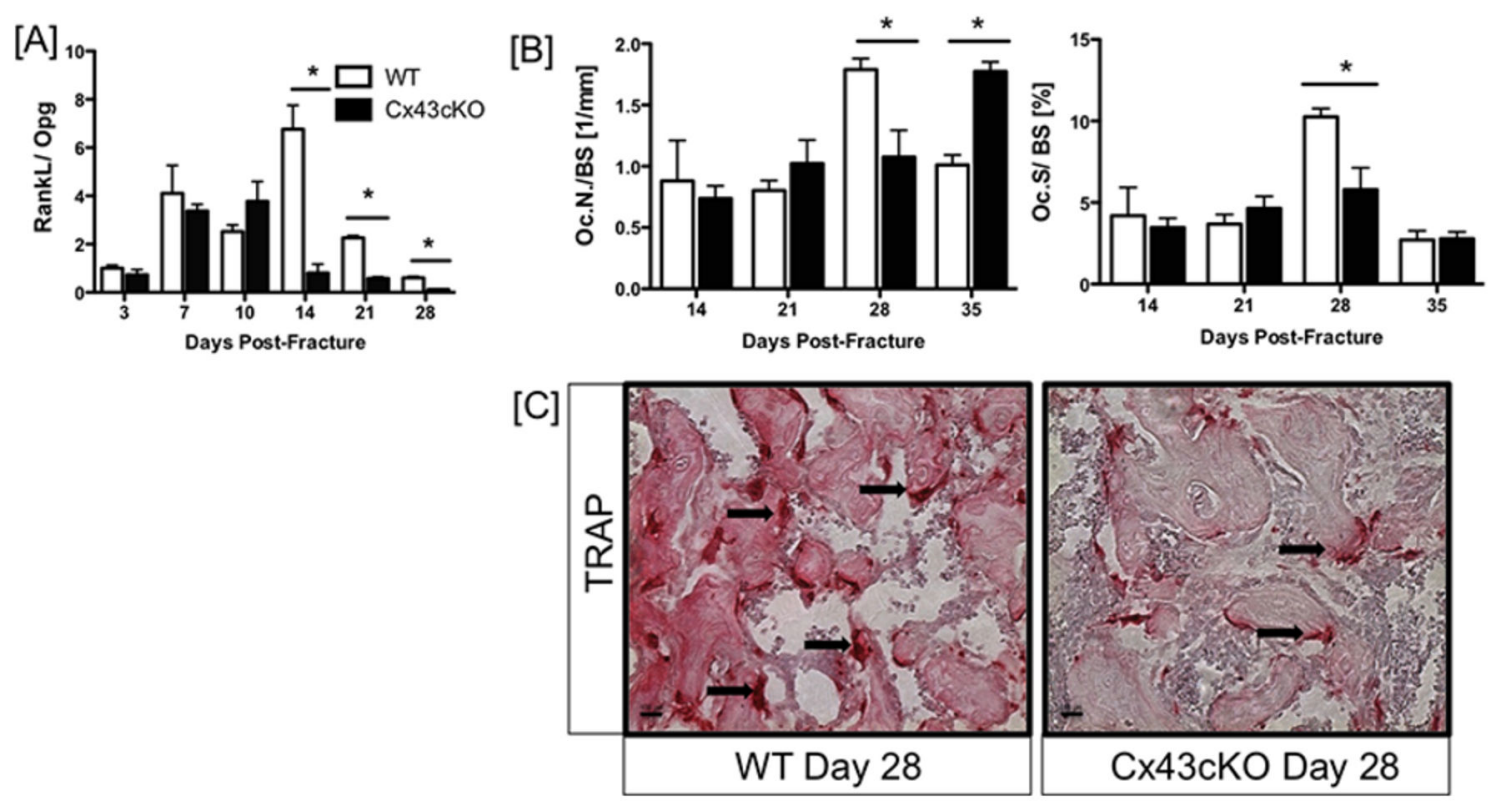
Impaired osteoclastogenesis in Cx43cKO fractures. [A] RankL/ Opg ratio calculated from qPCR analysis of RankL and Opg from tissue extracted from WT and Cx43cKO fracture calluses between 3-28 days. Tissue was pooled from 3 specimens/ genotype/ time-point, (*) indicates p<0.05 between WT and Cx43cKO at a given time-point. [B and C] Quantitative analysis of TRAP^+^ osteoclast number [B] and size [C] in WT and Cx43cKO fractures between 14-35 days. Cx43cKO fractures have significantly decreased Osteoclast number and Osteoclast surface at 28 days relative to WT. (*) indicates p<0.05 between groups at a given time-point, n=4 specimens/genotype/ time-point. [D] Representative images of TRAP stained sections of WT and Cx43cKO fractures at day 28 post-fracture. Black arrows indicate TRAP^+^ osteoclasts; scale bar represents 100μM.

In addition to alterations in chondrogenesis and bone formation, remodeling was also decreased in Cx43KO fractures, with significant decreases in RankL (*Tnsfs11*) expression between 14 and 28 days (Figure S1C). Peak RankL expression occurred at 14 days in WT (133-fold) and was significantly increased relative to Cx43cKO (2.26-fold, p<0.0001). RankL expression remained significantly elevated in WT fractures through 28 days relative to Cx43cKO. Interestingly, expression of the RankL decoy receptor Osteoprotegerin (Opg, *Tnsfs11b*) was not significantly different between WT and Cx43cKO fractures during healing, with peak expression in both groups at 28 days (WT: 209-fold, Cx43cKO: 210-fold, p>0.05) (Figure S1D). These results translate to a significant decrease in the RankL/Opg ratio between 14 and 28 days post fracture in Cx43cKO fractures (Day 14: 0.88-fold) relative to WT (Day 14: 6.7-fold, p<0.0001)(Figure 2A). The number and size of TRAP^+^ osteoclasts were quantified in the fracture callus of WT and Cx43cKO fractures between 14-35 days. No differences in Osteoclast number (Oc.N./BS) or Osteoclast size (Oc.S./BS) were observed between WT and Cx43cKO at 14 or 21 days. By 28 days, there was a significant decrease in both Oc.N./BS (WT: 1.79 ± 0.08; cKO: 1.075 ± 0.22, p=0.039) and Oc.S./BS (WT: 0.102 ± 0.005; cKO: 0.057 ± 0.01, p=0.036) in Cx43cKO fractures relative to WT. At day 35, no changes in Oc.S./ BS were observed, however, Oc.N./BS was significantly increased in Cx43cKO fractures (1.77± 0.13) relative to WT (1.01 ± 0.14, p=0.002) (Figure 2B).

### Functional deficits in healing Cx43cKO fractures

**Figure 3:**
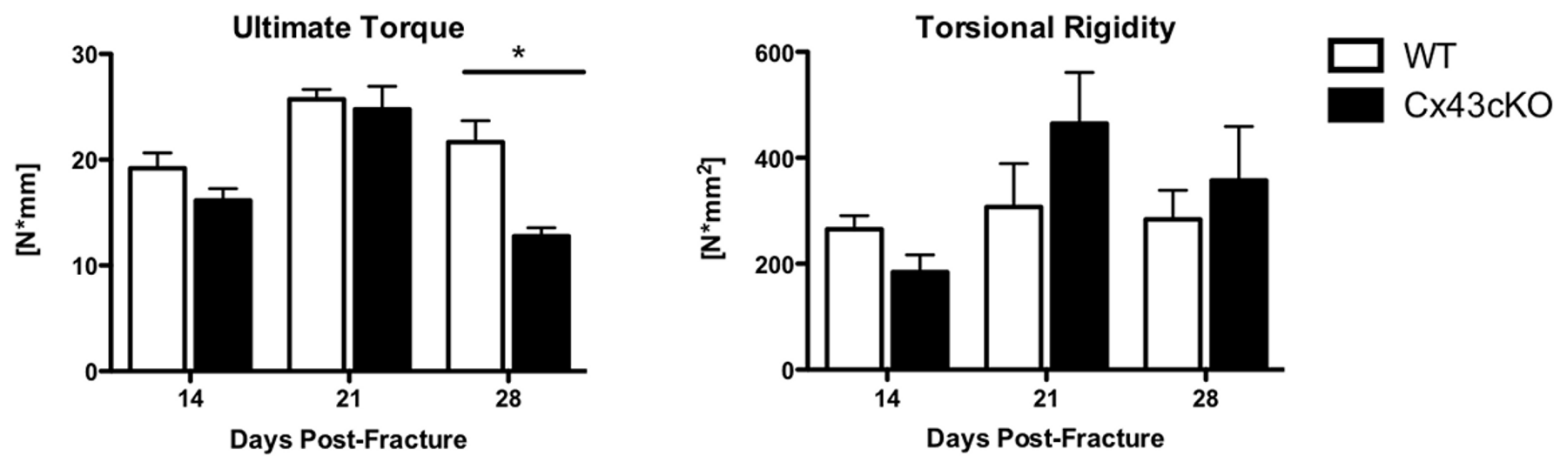
Functional deficits in healing Cx43cKO fractures. Ultimate Torque at Failure and Torsional Rigidity of WT and Cx43cKO fractures between 14-28 days. (*) Indicates p<0.05 between genotypes at a given time-point. N=8-10 genotype/time-point.

Ultimate torque at failure (T_ult_) was not significantly different between WT and Cx43KO fractures at 14 (WT: 19.18 ± 1.44 N*mm; Cx43cKO: 16.13 ± 1.12 N*mm, p=0.11) or 21 days post-fracture (WT: 25.7 ± 0.92 N*mm, Cx43cKO: 24.75 ± 2.19 N*mm, p=0.7). Peak T_ult_ occurred at 21 days in both WT and Cx43KO fractures. Cx43cKO fractures had significantly decreased T_ult_ at 28 days (12.77 ± 0.79 N*mm) compared to WT (21.66 ± 2.01 N*mm, p=0.009) (Figure 3). Torsional rigidity (T.R.), or stiffness, was not significantly different between WT and Cx43cKO fractures from 14 to 28 days (Day 14: WT: 265.4 ± 25.33 N*mm^3^, Cx43cKO: 184.1 ± 32.81 N*mm^3^, p=0.059), and peak T.R. occurred in both groups at 21 days (WT: 307.4 ± 81.89 N*mm^3^; Cx43cKO: 464.6 ± 96.78 N*mm^3^, p= 0.25) (Figure 3). 

### Attenuated β-catenin expression in Cx43cKO fractures

**Figure 4:**
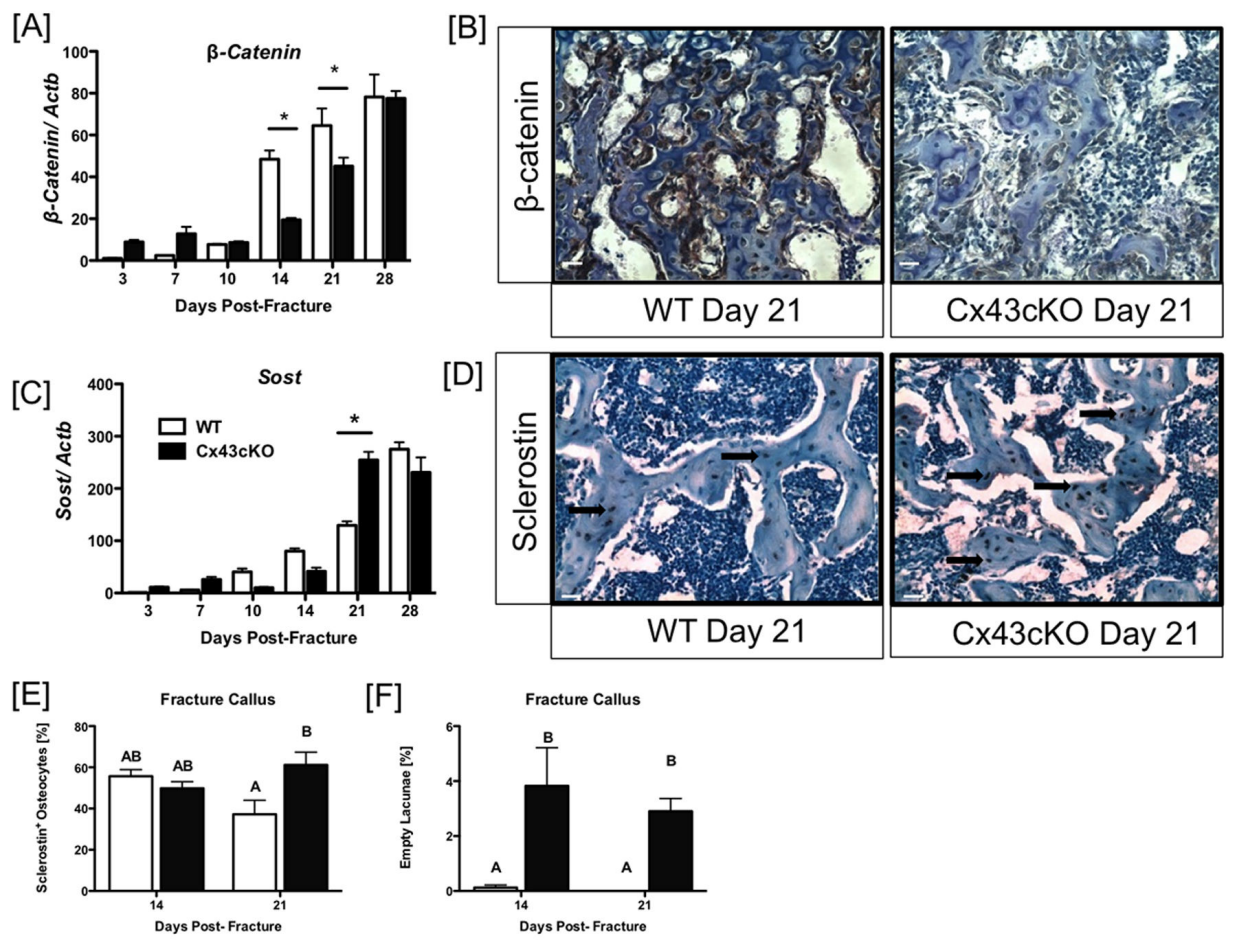
Attenuated β-catenin and enhanced Sclerostin expression in Cx43cKO fractures. [A -D] Quantitative and immunohistochemical expression of [A & B] β-catenin and [C &D] Sclerostin (Sost) in WT and Cx43cKO fracture calluses. Tissue for qPCR analysis was harvested between 3-28 days fracture. Tissue was pooled from 3 specimens/ genotype/ time-point, (*) indicates p<0.05 between WT and Cx43cKO at a given time-point. Immunohistochemistry images are 20x magnification from 21 days post-fracture; scale bar represents 100 microns. Arrows indicate Sclerostin^+^ cells. [E & F] Quantification of [E] Sclerostin^+^ osteocytes and [F] empty lacunae in the fracture callus of WT and Cx43cKO mice at 14 and 21 days. Different letters indicate p<0.05, while the same letter indicates p>0.05 between groups. n=3 specimens/ genotype/ time-point.

To explore possible mechanisms of decreased bone formation in Cx43cKO fractures, alterations in Wnt/β-catenin expression were examined. Expression of β-catenin, a regulator of bone formation was significantly increased in WT fractures relative Cx43cKO at 14 (WT: 48.4-fold; cKO: 19.4-fold, p<0.001) and 21 days post-fracture (WT: 64.5-fold, cKO: 45.1-fold, p<0.05) (Figure 4A). An observable decrease in β-catenin expression (by immunohistochemistry) occurred at 21 days in Cx43cKO fractures relative to WT (Figure 4B). Sclerostin (*Sost*), a negative regulator of bone formation that acts by inhibiting Wnt/β-catenin signaling, was significantly elevated in Cx43cKO fractures at 21 days (254-fold) relative to WT (129-fold, p<0.001) (Figure 4C). At 14 days post-fracture the number of Sclerostin^+^ osteocytes was not significantly different between WT and Cx43cKO fractures (p>0.05), however, by 21 days, the number of Sclerostin^+^ osteocytes was significantly increased in Cx43cKO fractures (61.16 ± 10.7) relative to WT (37.23 ± 11.78, p<0.05) (Figure 4D & E). There was no difference in the number of Sclerostin^+^ osteocytes in the cortical bone (proximal/distal or callus adjacent) between WT and Cx43cKO during fracture healing (Figure S2B). Consistent with a previously reported phenotype of increased osteocyte apoptosis in Cx43cKO bones [9], there was a significant increase in the number of empty osteocyte lacunae in the callus of Cx43cKO fractures relative to WT at both 14 (WT: 0.116 ± 0.16 , cKO: 3.82 ± 2.4, p<0.05) and 21 days post-fracture (WT: 0 ± 0, cKO: 2.9 ± 0.8, p<0.05) (Figure 4E). No significant differences in the number of empty lacunae were observed in the proximal/distal cortical bone or cortical bone adjacent to the fracture site between WT and Cx43cKO at 14 and 21 days (Figure S2C). 

**Figure 5:**
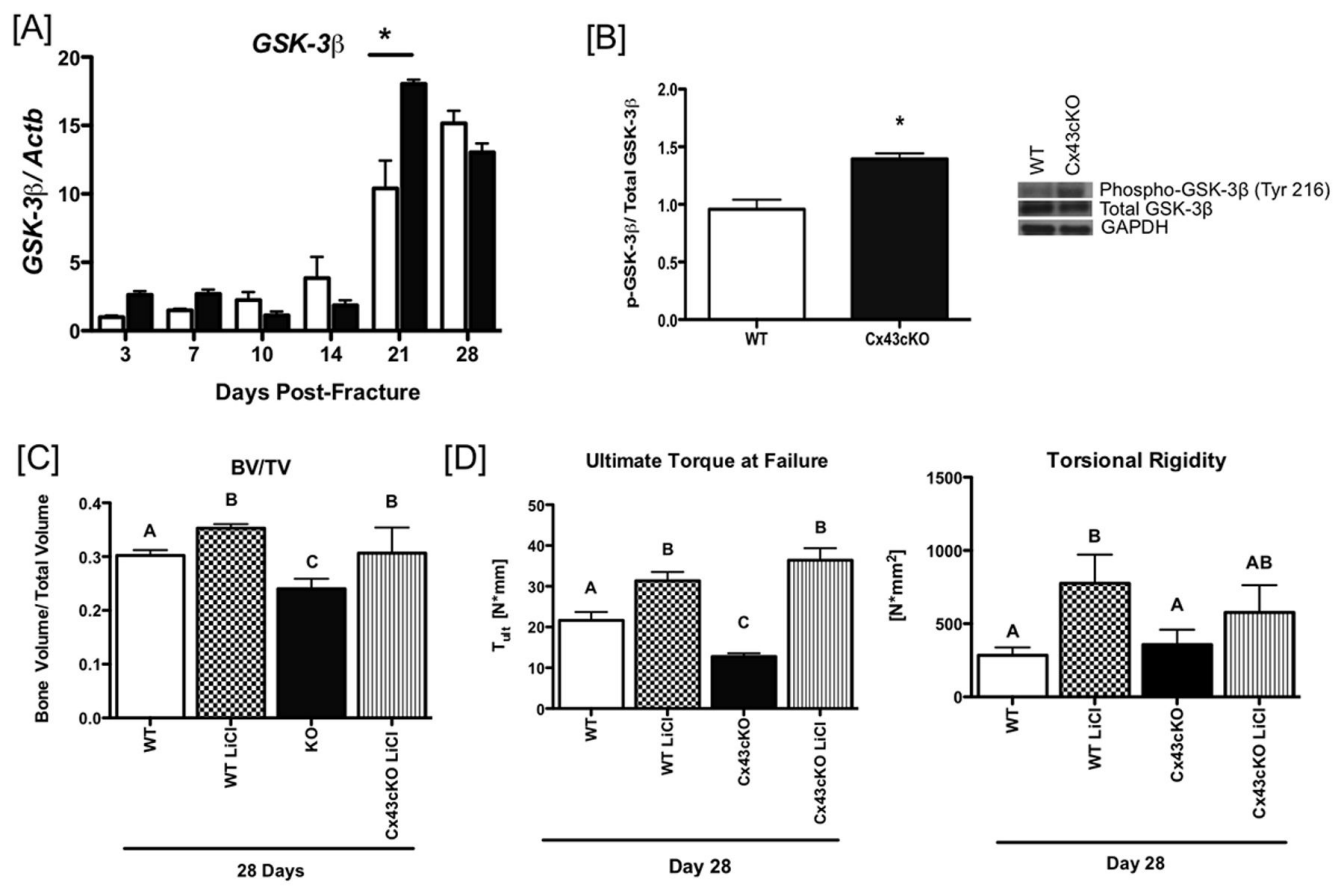
Inhibition of GSK-3β restores normal bone formation in Cx43cKO fractures. [A] Expression of *GSK-3*β in tissue extracted from WT and Cx43cKO fracture calluses between 3-28 days. Tissue was pooled from 3 specimens/ genotype/ time-point, (*) indicates p<0.05 between WT and Cx43cKO at a given time-point. [B] Expression of active GSK-3β (Tyr 216), total GSK-3β and GAPDH protein in WT and Cx43cKO fractures at 21 days. N=3 specimens/ genotype. [C] Bone volume/ total volume (BV/TV) of WT and Cx43cKO fractures treated with LiCl. Different letters indicate p<0.05, while the same letter indicates p>0.05 between groups. n=5-7 specimens/genotype/ time-point. [D and E] Ultimate torque at failure [D] and torsional rigidity [E] of WT and Cx43cKO fractures treated with LiCl. Different letters indicate p<0.05, while the same letter indicates p>0.05 between groups. n=5-7 specimens/genotype/ time-point.

 GSK-3β is a key component of the β-catenin proteasomal degradation complex and its activity is determined by specific phosphorylation events. GSK-3β mRNA expression was significantly increased in Cx43cKO fractures at 21 days (18-fold) relative to WT (10.4-fold, p<0.0001) (Figure 5A). A significant increase in the active form (Phosph-Tyr216) of GSK-3β protein was observed in Cx43cKO (1.46-fold vs. WT, p=0.01) fractures relative to WT at 21 days (Figure 5B).

 To determine if changes in β-catenin expression in Cx43cKO fractures were the result of increased GSK-3β activity, we attempted to rescue the bone formation phenotype associated with Cx43 deficiency by inhibiting GSK-3β activity using LiCl. LiCl treatment resulted in a significant increase in BV/TV at 21 and 28 days relative to respective genotype controls not treated with LiCl. At 28 days, BV/TV in WT fractures treated with LiCl (0.35 ± 0.007) was significantly increased relative to un-treated WT (0.30 ± 0.01, p=0.015), while Cx43cKO fractures treated with LiCl (0.31 ± 0.02) were significantly increased compared to untreated Cx43cKO fractures (0.24 ± 0.015, p=0.04). Importantly, there was no longer a significant decrease in callus BV/TV in Cx43cKO fractures treated with LiCl compared to LiCl treated WT fractures (WT: 0.35 ± 0.007; Cx43cKO: 0.31 ± 0.02, p=0.19), suggesting that inhibition of GSK-3-β activity can restore normal bone formation in Cx43cKO fractures (Figure 5C, Figure S3). In addition to BV/TV, inhibition of GSK-3β rescued the impaired biomechanical properties associated with Cx43cKO fractures. LiCl treatment increased T_ult_ in both WT and Cx43cKO fractures relative to un-treated genotype controls at 28 days. There was no significant difference in T_ult_ in LiCl treated Cx43cKO fractures (36.4 ± 5.83 N*mm) relative to LiCl treated WT fractures (43.56 ± 12.22 N*mm, p=0.58) at 28 days. Torsional rigidity was significantly increased in WT fractures treated with LiCl (776.1 ± 276.3 N*mm^2^) relative to un-treated WT fractures (283.8 ± 145.1 N*mm^2^, p=0.008) at 28 days. No change in T.R. was observed between WT and Cx43cKO fractures treated with LiCl (Figure 5D). 

## Discussion

In this study we have demonstrated that loss of Cx43 in osteoblasts and osteocytes using Col1-Cre results in delayed osteoblastic differentiation, decreased bone formation and impaired mechanical properties. We further show that healing deficits in Cx43cKO fractures may be the result of attenuated Wnt/β-catenin signaling due to increased Sclerostin expression or GSK-3β activity. These findings suggest that Cx43 plays an important role in restoration of normal bone architecture and mechanical strength during fracture healing by modulating β-catenin signaling.

The use of the Col1-Cre results in significant decreases in *Gja1* expression in Cx43cKO fractures relative to WT between 10-28 days, but no differences were observed at 3 or 7 days post-fracture. These findings are likely due to the specific cellular composition of the fracture callus during each stage of healing. Early inflammatory events are characterized by the presence of inflammatory cells including macrophages, platelets and lymphocytes [32], many of which express Cx43 [33,34], however, changes in Cx43 expression in inflammatory cells would not be expected using the Col1-Cre [35]. 

Alterations in healing are more pronounced during the mineralization phase of healing as the cartilaginous callus is replaced with areas of new mineralized tissue due to increased osteoblastic differentiation and activity [32]. Impaired bone formation during healing in Cx43cKO fractures is consistent with an important role for Cx43 in osteoblastic differentiation. *In vitro*, Cx43 deficiency impairs osteoblast maturation [15-17,36], with Cx43 knock-down resulting in almost complete abrogation of Alkaline Phosphatase (AP) activity [17]. Additionally, a microRNA that targets Cx43 inhibits differentiation, resulting in a decrease in both AP activity and *Bglap* expression, both of which can be rescued by restoring Cx43 expression [36]. *In vivo*, osteopenia is observed in bone cell-specific Cx43 deficient mice [10,18,19] and in mice with complete loss of Cx43 [37]. 

Consistent with impairments in osteogenesis, peak expression of *Col1a1*, Oc and *Bmp2* were delayed in Cx43cKO fractures relative to WT, while a significant decrease in BV/TV occurred at 14 and 28 days in these mice. Mineralization of the callus occurs in two stages during fracture healing: primary and secondary bone formation [38]. In WT fractures, increased MAR, an index of how quickly osteoblasts form and mineralize new bone, and BFR, which is a measure of the volume of mineralized bone formed per unit time, occurred at 21 days and coincides with peak Osteocalcin expression during the primary bone formation stage. This period of robust bone formation results in a mineralized callus and is followed by an increase in osteoclastic bone resorption at 28 days. Secondary bone formation encompasses long-term remodeling of the bone to restore normal architecture. In WT fractures the secondary bone formation stage is characterized by increases in MS/BS and BFR relative to Cx43cKO at 35 days. Primary bone formation occurs in Cx43cKO fractures, however, delays in osteogenesis shift the time-frame in which these events occur and lead to morphological and functional deficits relative to WT at a given time, including decreased bone volume fraction and T_ult_ at 28 days. Support for delayed, rather than impaired, osteogenesis is provided by the increased BFR at 28 days relative to WT, along with peak Osteocalcin expression. A second increase in bone formation is not observed in Cx43cKO fractures because time-points after 35 days were not analyzed. Delays in osteogenesis in Cx43cKO fractures impact subsequent bone resorption and remodeling, consistent with decreased osteoclast number and size relative to WT at 28 days. Furthermore, active remodeling in Cx43cKO fractures at 35 days is suggested by the increase in osteoclast number relative to WT at this time. 

This study supports the emerging concept that Cx43 is involved in regulating bone formation by modulating the Sclerostin/Wnt/ β-catenin signaling axis [9,19,39,40]. However, the mechanisms through which this interaction occurs and the resultant effects on bone formation differ markedly between fracture healing and the response to mechanical loading. 

Decreased Sclerostin expression has been observed in cortical bone of Cx43 deficient mice [9,19,40]. Bivi et al showed that Cx43 deficiency decreases Sclerostin expression in osteocyte specific Cx43cKO mice [40], a finding we have recently confirmed in osteoblast/osteocyte specific Cx43 deficient mice [9]. This would seem to suggest that Cx43 deficiency would lead to enhanced β-catenin expression and robust bone formation, however, the osteopenic phenotype in Cx43 deficient mice is well documented [10,18,19]. Recently, Bivi and colleagues reported increased β-catenin expression in an osteocyte-enriched population of cells from Cx43 deficient ulnae, suggesting that Cx43 deficiency leads to an accumulation of free β-catenin, and as such bone cells are ‘primed’ to respond to mechanical signals [39]. These data suggest the involvement of Cx43 in a negative feedback loop to regulate the bone cell response to anabolic stimuli and are consistent with the enhanced responsiveness to mechanical loading that occurs in Cx43 deficient mice [10,41]. In contrast, we observed increases in *Sost* mRNA and the number of Sclerostin^+^ cells in the callus of Cx43cKO fractures at 21 days, which coincides with a significant attenuation of β-catenin expression and MAR. Interestingly, these findings are supported by data from Grimston et al in a model of high strain mechanical loading. Col1-cre mice were loaded at a strain of 4665 με, which results in an injury response and formation of woven bone rather than lamellar bone formation that normally occurs during anabolic loading. At this strain, decreased response to mechanical loading was observed in Cx43 deficient mice [6], indicating that the role of Cx43 in osteogenesis differs in the contexts of anabolic loading and response to injury. 

In contrast to Sclerostin, which indirectly attenuates β-catenin expression by inhibiting the interaction of Wnt ligands with the low-density lipoprotein receptor-related protein 5 or 6 (Lrp5/6) and frizzled receptor complex, GSK-3β can directly control β-catenin activity. Phosphorylation of β-catenin by GSK-3β results in proteasomal degradation and inhibition of transcription of β-catenin responsive osteogenic genes. In turn, the activity of GSK-3β is determined by specific phosphorylation events; phosphorylation at Ser9 results in in-activation of GSK-3β toward many substrates, while phosphorylation at Tyr216 is activating [42]. The link between Cx43 and GSK-3β is unclear, however, preliminary studies suggest that loss of Cx43 decreases phosphorylation of GSK-3β at Ser9 thus increasing GSK-3β activity [43]. Here we show an increase in active GSK-3β in Cx43cKO fractures and restoration of normal healing when GSK-3β activity is inhibited by LiCl treatment in Cx43cKO fractures. These data suggest that Cx43 can regulate bone formation during fracture healing by modulating the phosphorylation state and activity of GSK-3β, resulting in attenuated β-catenin expression in the case of Cx43 deficiency. 

In summary, we have shown that targeted deletion of Cx43 in osteoblasts/ osteocytes delays bone formation and impairs restoration of mechanical properties during healing. Furthermore, our data suggests that Cx43 can alter the activity of GSK-3β and therefore β-catenin expression during fracture healing, identifying Cx43 as a key player in bone formation during fracture repair. To our knowledge this is the first examination of the effects of Cx43 during fracture healing with a specific focus on the mechanisms by which healing is impaired. These data support Cx43 as an important therapeutic target to enhance fracture healing and suggest that targeted overexpression of Cx43 may improve fracture healing.

## Supporting Information

Figure S1 **MicroCT and qPCR analysis of WT and Cx43cKO fractures.** [A] Bone volume (BV) and [B] Total volume (TV) of WT and Cx43cKO fractures between 14-35 days. (*) indicates p<0.05 between WT and Cx43cKO fractures at a given time-point. Expression of [C] RankL (Tnsfs11), and [D] Opg (Tnsfs11b) in tissue extracted from WT and Cx43cKO fracture calluses between 3-28 days. Tissue was pooled from 3 specimens/ genotype/ time-point, (*) indicates p<0.05 between WT and Cx43cKO at a given time-point.
(TIF)

Figure S2 **Quantification of Sclerostin^+^ and empty lacunae.** [A] Cortical bone segmented into proximal/distal/callus adjacent areas. Quantification of [B] Sclerostin^+^ osteocytes and [C] empty lacunae in the proximal/distal segments of cortical bone of WT and Cx43cKO mice at 14 and 21 days. n=3 specimens/ genotype/ time-point.
(TIF)

Figure S3 **Bone volume (BV) and Total volume (TV) of WT and Cx43cKO fractures treated with LiCl and harvested at 21 and 28 days post-fracture.** Different letters indicate significant difference (p<0.05), while the same letter indicates p>0.05, n=5/genotype/ time-point.
(TIF)
