# Supplementary figures and images for: Inhibition of GSK-3β Rescues the Impairments in Bone Formation and Mechanical Properties Associated with Fracture Healing in Osteoblast Selective Connexin 43 Deficient Mice
Source: PLoS One. 2013 Nov 8;8(11):e81399. doi: 10.1371/journal.pone.0081399 (PMC3832658; doi:10.1371/journal.pone.0081399)

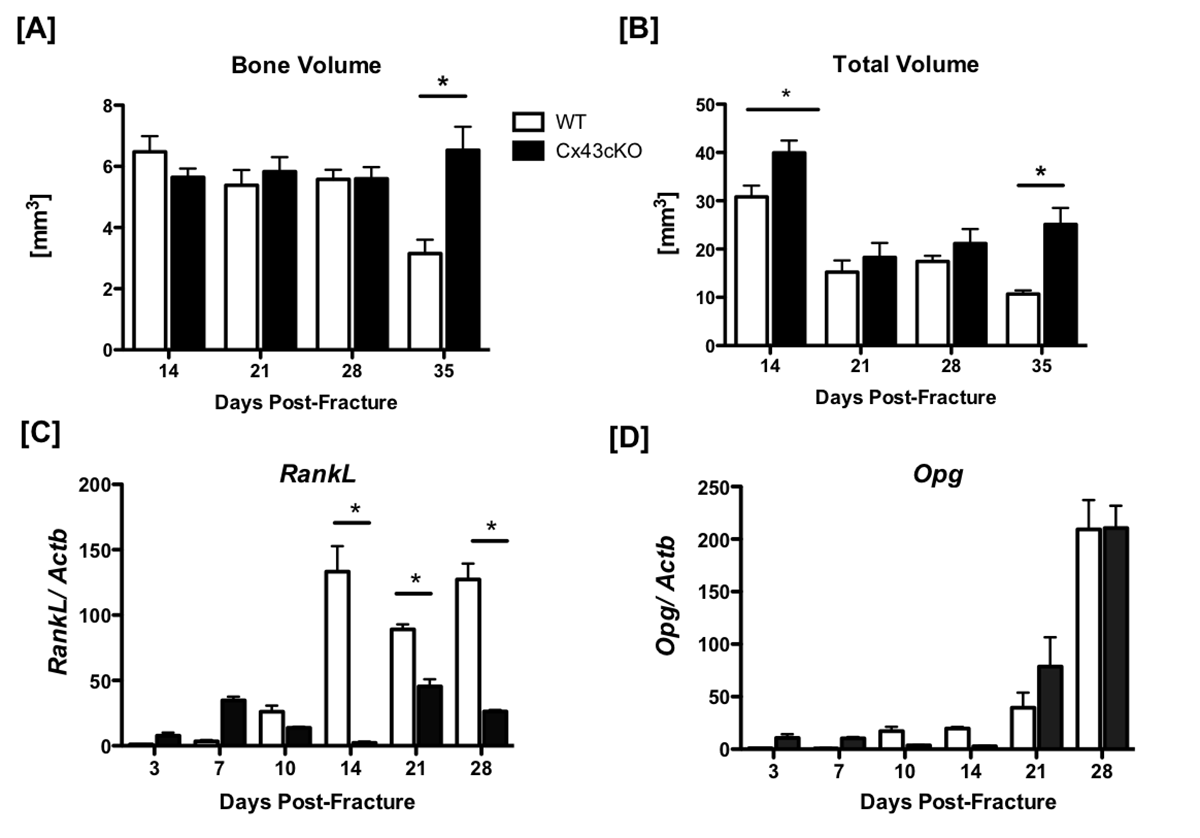

Supplement: Figure S1 — MicroCT and qPCR analysis of WT and Cx43cKO fractures. [A] Bone volume (BV) and [B] Total volume (TV) of WT and Cx43cKO fractures between 14-35 days. (*) indicates p<0.05 between WT and Cx43cKO fractures at a given time-point. Expression of [C] RankL (Tnsfs11), and [D] Opg (Tnsfs11b) in tissue extracted from WT and Cx43cKO fracture calluses between 3-28 days. Tissue was pooled from 3 specimens/ genotype/ time-point, (*) indicates p<0.05 between WT and Cx43cKO at a given time-point. (TIF) [file pone.0081399.s001.tif]

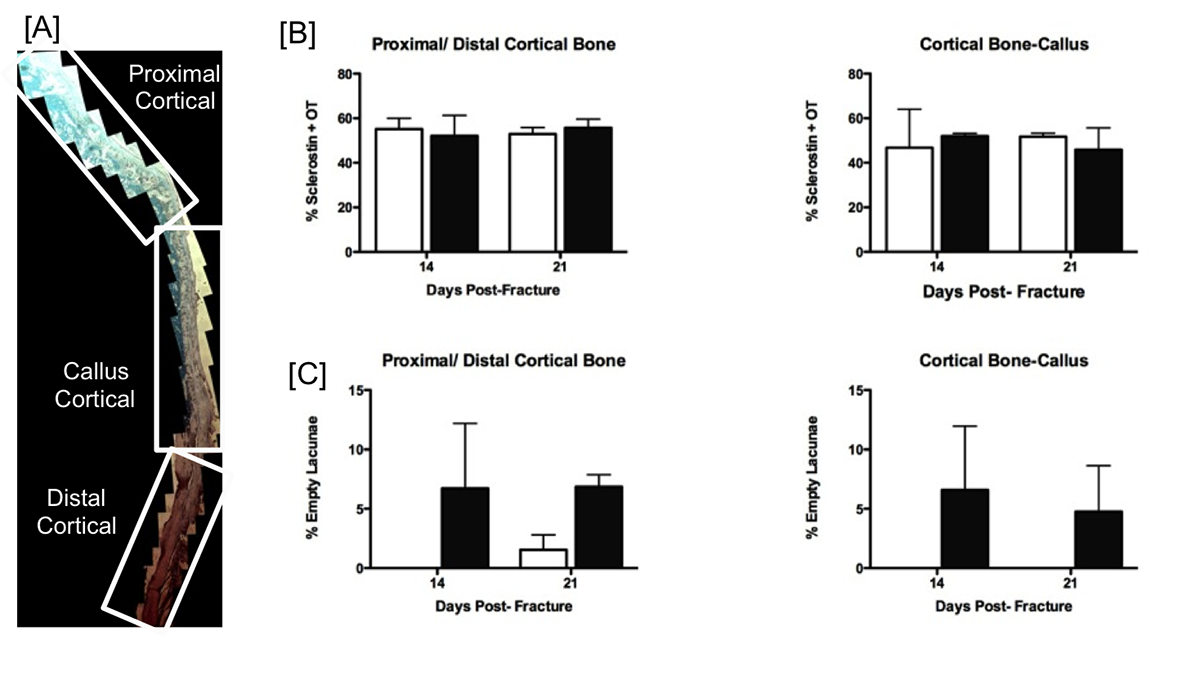

Supplement: Figure S2 — Quantification of Sclerostin+ and empty lacunae. [A] Cortical bone segmented into proximal/distal/callus adjacent areas. Quantification of [B] Sclerostin+ osteocytes and [C] empty lacunae in the proximal/distal segments of cortical bone of WT and Cx43cKO mice at 14 and 21 days. n=3 specimens/ genotype/ time-point. (TIF) [file pone.0081399.s002.tif]

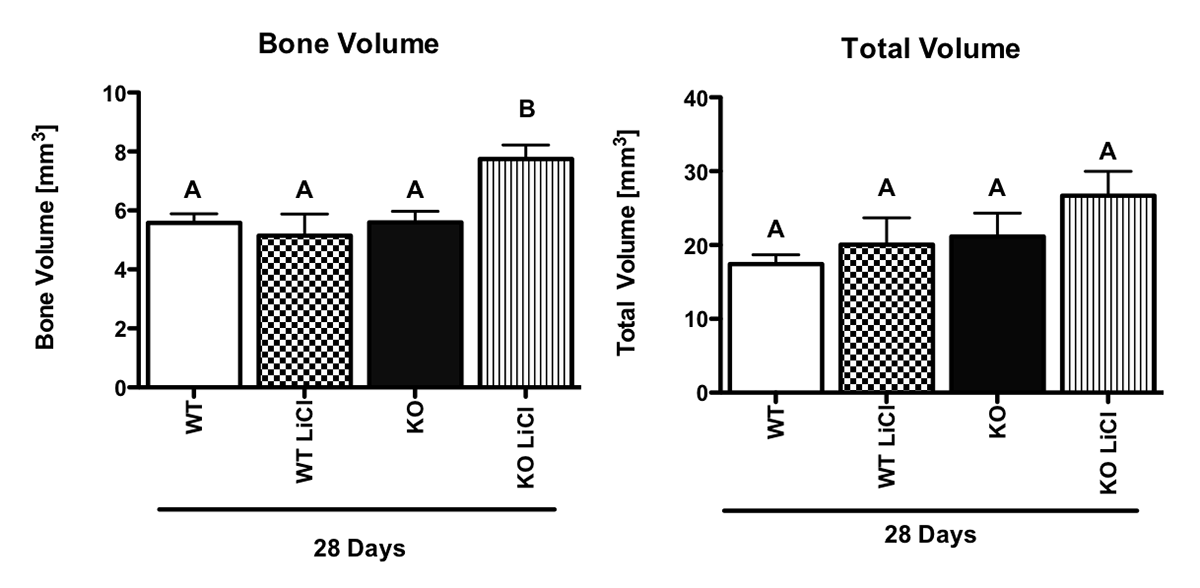

Supplement: Figure S3 — Bone volume (BV) and Total volume (TV) of WT and Cx43cKO fractures treated with LiCl and harvested at 21 and 28 days post-fracture. Different letters indicate significant difference (p<0.05), while the same letter indicates p>0.05, n=5/genotype/ time-point. (TIF) [file pone.0081399.s003.tif]
